# Better communication between experts is needed to solve the environmental origins of birth defects

**DOI:** 10.1002/bies.202100241

**Published:** 2021-11-30

**Authors:** Duncan B. Sparrow

**Affiliations:** ^1^ Department of Physiology, Anatomy and Genetics University of Oxford Oxford UK

**Keywords:** animal models, birth defects, developmental biology, environmental risk factors, epidemiology, interdisciplinary research, teratology

## Abstract

More than 6% of babies are born with a structural or functional defect, and many of these need special care and treatment to survive and thrive. Such defects can be inherited, arise through exposure to altered conditions or compounds in the womb, or result from a combination of genetic and environmental factors. Since the 1940s, animal experiments and epidemiological studies have identified many environmental factors that can cause particular birth defects. More recently, advances in genomics have allowed a simple genetic diagnosis in ∼ 30% of birth defects. However, the cause of the remainder is a mystery. I believe that a key limiter to successful identification of new environmental factors is that clinicians, epidemiologists and developmental biologists all approach the topic from different angles. I propose that better communication between such experts will further increase our understanding of the environmental causes of birth defects, and potentially reduce their global burden.

## Graphical abstract

About 6% of babies are born with a birth defect. Historically, many environmental risk factors causing these defects have been identified. However, this research has stalled in recent years. I believe that the time is now right to re‐invigorate this field through closer collaboration between clinicians, epidemiologists and developmental biologists.

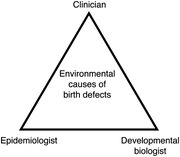

## INTRODUCTION

Birth defects are a major global problem, affecting ∼ 6% of live births, and more than 10% of miscarriages.^[^ ^1^ ^]^ Many of these result in infant mortality, and many of those who survive face lifelong disability and treatment. The burden of mortality is particular high in middle‐ and low‐income countries, where perinatal medical interventions and on‐going treatments may not be routinely available. Since ancient times, people have speculated about the causes of birth defects, discussing the various merits of inherited, environmental or supernatural causes. The latter two explanations were in vogue until the late 19th century, when the rediscovery of Gregor Mendel's work shifted researcher attention to genetics. This focus has largely remained to the present day, especially over the past 30 years with the revolution in genetic and genomic technologies. As a result, simple genetic causes have been found for many types of birth defect, and sequence variants in hundreds of genes have been associated with individual cases in particular families. Such genetic diagnosis is incredibly useful for individual families to provide information on issues such as prognosis, treatment and recurrence rate. However, understanding the genetic causes of birth defects has had relatively little impact on the population prevalence of birth defects. This is because in most cases the causative sequence variants are unique to each family. Thus, beyond identifying candidate genes for sequencing in new patients, this knowledge has limited applicability to other families or the general population. Furthermore, even with the great success of genetic diagnosis, the causes of two‐thirds of birth defects remain unknown. This is because birth defects can also be caused by environmental factors that cause altered conditions in the womb during embryonic development. This can be due to alteration of maternal physiology, or exposure of the developing embryo to teratogenic compounds. In contrast to genetic studies, identification of such environmental risk factors has scope for reducing the population prevalence of these birth defects. Despite this great potential, studies of the environmental causes of birth defects have stalled in recent years. This is despite the great advancements of our understanding of human embryogenesis, and the increasing use of electronic health records in primary health care. Even though an interdisciplinary approach may be self‐evident, I believe that the time is now right to re‐invigorate this field of research through closer collaboration between clinicians, epidemiologists and developmental biologists.

In general, the association of particular environmental factors with specific birth defects can be identified in two ways, either via experimental teratology in animal models, or by epidemiology of human populations.

## EXPERIMENTAL TERATOLOGY USES ANIMAL MODELS TO DISCOVER RISK FACTORS CAUSING BIRTH DEFECTS

Mammalian embryonic development is a complex process that cannot be easily modelled in vitro. For this reason, the best way to test whether a particular environmental factor can cause birth defects is by animal experimentation. Early studies took a sledgehammer approach, exposing pregnant animals to different compounds or environmental conditions, then examining the effects on the resultant offspring. Animal studies are still used today when new therapeutic compounds are in the process of being approved for use in humans (*ICH S5 (R3) guideline on reproductive toxicology: Detection of toxicity to reproduction for human pharmaceuticals*. (2020). Retrieved from https://www.ema.europa.eu/en/ich‐s5‐r3‐guideline‐reproductive‐toxicology‐detection‐toxicity‐reproduction‐human‐pharmaceuticals#current‐version‐section). This is because reliable in vitro models for embryogenesis have not been developed.^[^ ^2^ ^]^ However, these studies have become increasingly refined. In the last 30 years, developmental biology has made great strides in understanding how and when many tissues and organs are formed during embryogenesis. In addition, many of the underlying molecular mechanisms have been identified. This knowledge is now being used to plan new studies, as well as re‐examine the findings of old teratological studies. For example, in the 1950s exposure of pregnant mice to lowered oxygen levels was shown to cause malformation of the embryo's heart and vertebrae.^[^ ^3^ ^]^ 60 years later, the application of modern molecular techniques including candidate gene analysis and untargeted transcriptomics showed that the lack of oxygen perturbs the Fibroblast growth factor (FGF) signaling pathway in the developing embryo to cause the defects.^[^ ^4^, ^5^ ^]^

## EPIDEMIOLOGY STUDIES HUMAN POPULATIONS TO DISCOVER RISK FACTORS CAUSING BIRTH DEFECTS

Epidemiology takes large populations of people, and compares the exposures to potential risk factors of cases with a birth defect and matched controls who lack the defect. Environmental factors for analysis by epidemiology are generally selected in one of two ways. Firstly, anecdotal observation of increased rates of birth defects in a small population can be used to generate a testable hypothesis. One of the first examples of using this approach to identify an environmental factor causing birth defects is maternal infection with the *Rubella* virus in the first trimester. The anecdotal association of maternal *Rubella* infection with a specific suite of offspring birth defects was first described Norman Gregg in 1941.^[^ ^6^ ^]^ However, it took some years for the scientific and medical communities to accept his hypothesis, as at the time it was considered that only inherited factors could cause birth defects. Secondly, environmental teratogens identified through animal studies can be verified by epidemiology. One of the best known examples of this strategy is maternal folate deficiency. This was first linked to birth defects through experiments in rats more than 60 years ago by Margaret Nelson and Herbert Evans (co‐discoverer of Vitamin E and inventor of ‘Evans blue’ dye).^[^ ^7^ ^]^ This was confirmed in the 1980s by several large‐scale epidemiological studies, and finally in 1990 a large scale clinical trial proved conclusively that maternal folate supplementation significantly reduced the risk of having a child with a neural tube defect.^[^ ^8^ ^]^

## TRANSLATION OF RESEARCH TO REDUCE BIRTH DEFECT PREVALENCE IS NOT STRAIGHTFORWARD

Once an environmental risk factor has been identified, this knowledge can be translated by either avoidance or by mitigation. The simplest approach to minimize the risk of having a child with a birth defect is to simply avoid exposure to environmental teratogens. However, in practice this may be more complicated than it first appears. Firstly, to avoid a risk factor, it must be identified in advance of the pregnancy. Although dozens of environmental risk factors are known or suspected, it is likely that yet others remain to be discovered, and these cannot be avoided. Secondly, half of all pregnancies in developed countries are unintended.^[^ ^9^ ^]^ Therefore, public health education can only be effective if it leads to all women of child‐bearing age actively avoiding environmental risk factors. Thirdly, therapeutic drugs with well‐known teratogenic side effects ideally should not be used to treat pregnant women. However, in some cases the health benefits to the mother may be judged to outweigh the risks of offspring birth defects. A prominent example of this is valproic acid (VPA), which is used as an anti‐seizure medication for epilepsy and increasingly for bi‐polar syndrome. Withdrawal of VPA during pregnancy can have dramatic and life‐threatening effects on the mother. Lastly, avoidance of some risk factors requires active public health intervention. For example, it is well‐documented that maternal infection with the *Rubella* virus in early pregnancy carries a high risk of offspring birth defects.^[^ ^10^ ^]^ Population vaccination is an effective way of limiting the chances of *Rubella* exposure during pregnancy. However, for *Rubella* ∼ 90% of the entire population needs to be vaccinated to provide sufficient herd immunity to prevent disease outbreaks. Sadly, growth of the anti‐vaccination movement in some developed nations in recent years has led to a resurgence of *Rubella*. Some environmental risk factors, such as maternal disease or altered physiology, cannot be avoided. Instead, the relative risk can be reduced by maternal behavior change. A good example is maternal pre‐existing type 1 or type 2 *diabetes mellitus* (PGDM). It is well‐established that PGDM substantially increases the risk of having a child with one or more specific types of birth defect.^[^ ^11^ ^]^ The teratogen is likely to be hyperglycemia itself, with offspring defect rate increasing with increasing maternal glycated hemoglobin (Hb1ac) levels, a routine measure of glycemic control. Fittingly, pre‐pregnancy care by specialist health professionals to improve maternal glycemic control has been shown to significantly reduce the risk of offspring birth defects.^[^ ^12^ ^]^ Finally, the effects of some environmental risk factors can be overcome by dietary alteration or medical intervention. The best known example is folic acid supplementation. It is now well known amongst the general public that folic acid deficiency increases the risk of having a child with a neural tube defect. This can be readily corrected by peri‐conceptional maternal folic acid supplementation, as shown in a landmark clinical trial published in 1990.^[^ ^8^ ^]^ Subsequently, changes in public health policy to recommend folate supplements for pregnant women, and/or mandated addition of folate to basic foodstuffs, has reduced the population prevalence of neural tube defects (NTDs) by up to 70%.

In conclusion, understanding of how each particular environmental risk factor causes birth defects can be used to design strategies to reduce the global incidence of birth defects. However, to achieve this aim most efficiently, cross‐disciplinary collaboration is needed. Unfortunately, communication between clinicians, epidemiologists and developmental biologists is not as good as it could be. Below, I summarize some of the most common misunderstandings between the different fields.

## WHAT THE CLINICIAN WISHES THE DEVELOPMENTAL BIOLOGIST AND THE EPIDEMIOLOGIST KNEW

It is essential to accurately describe the precise anatomy of an individual with a structural birth defect. This may seem pedantic, but simplification of description can lead to erroneous assumptions about the embryological origins of the defect. For example, congenital heart disease is not a single entity. The nomenclature suggested by the International Pediatric and Congenital Cardiac Code contains no less than 318 distinct entries,^[^ ^13^ ^]^ each potentially with a different embryological origin. However, many researchers investigating animal models of congenital heart disease (CHD) have an inadequate knowledge of normal embryonic heart morphology and do not accurately or comprehensively describe abnormal anatomy. For example, when using mouse models of birth defects, embryos are often described as having a “ventricular septal defect” (VSD). However, subtypes of VSDs include peri‐membranous, muscular, inlet, outlet, or as part of a common atrioventricular junction.^[^ ^14^ ^]^ If the precise subtype of VSD is identified, this may give important clues to the embryological origins of these defects.

***Solution***: To maximize the translational potential of basic research, developmental biologists should ask a clinical anatomist to assist them to accurately determine and describe the embryonic phenotypes in their animal models.

Likewise, some epidemiological studies test for an association between a particular environmental factor and the broad term “CHD”. However, both embryological and molecular origins of different types of heart defects can vary considerably. In addition, if smaller sub‐groups of CHD are used, these may not be grouped on the basis of embryological or molecular origin, but on final phenotype. Any particular environmental teratogen may well only perturb one particular process of embryonic heart development, and thus might only increase the risk of a few subtypes of CHD.

***Solution***: Epidemiologists should consult developmental biologists and clinical anatomists to identify sub‐groups of defects likely to have a similar embryological or molecular origin. Although, separating case groups into smaller sub‐types of defects will reduce case numbers, paradoxically this may well increase statistical power to detect risk factors.

## WHAT THE DEVELOPMENTAL BIOLOGIST WISHES THE CLINICIAN AND THE EPIDEMIOLOGIST KNEW

Most organs and structures in the human embryo form very early in gestation, over a short defined period. For example, in human embryos, the heart forms between weeks 3–8 of embryogenesis (weeks 5–10 after the last menstrual period). By the end of this period, the architecture of the embryonic heart is almost identical to that of an adult. Thus, there is only a short window in which the developing heart is susceptible to external factors to cause structural heart defects.

One of the most important issues in designing animal studies is when during embryogenesis the analysis is performed. This requires a detailed knowledge of basic developmental biology. Exposure to an environmental teratogen will have more than one effect on a developing embryo. The initial exposure is likely to have an immediate effect. Here, changes in the expression of specific genes, proteins, or signaling pathways can lead to altered cell metabolism and physiology, proliferation rate, migration or polarity. However, these initial effects generally do not cause immediately visible changes in the embryo. In many cases, any morphological changes will only become apparent at a later time, culminating in a structural or functional defect. Thus, the aim of any molecular investigation should be to analyses the embryos at, or immediately after, the teratogenic insult. However, many studies examine changes in gene, protein or signaling pathway expression when an altered phenotype has become apparent in the developing embryo. This likely to be long after the original molecular events causing altered embryogenesis have occurred. Therefore, these analyses will only identify the ***effects*** of altered development, rather than the underlying ***causes***. Furthermore, a lack of understanding of embryonic development and metabolism can lead to poor hypothesis building. One particularly prominent example is the case of diabetic embryopathy. As mentioned above, pre‐existing maternal diabetes carries a significant risk of having a child with one or more severe birth defects.^[^ ^11^ ^]^ This phenomenon has been studied for many years in animal models. One particularly popular hypothesis is that increased glucose uptake causes “increased reactive oxygen species (ROS)” in the developing embryo, and that this leads to specific effects on organ formation.^[^ ^15^ ^]^ It is certainly true in adult tissues that hyperglycemia causes elevated ROS. Indeed, this has been proposed as being the underlying common pathogenic mechanism for all adult diabetic complications.^[^ ^16^ ^]^ This is because excess glucose levels lead to an overproduction of superoxide in the mitochondrial electron transport chain during oxidative phosphorylation. However, in most tissues of the mouse embryo oxidative phosphorylation does not begin until after the chorioallantoic branching (CB) stage (∼E9.5), when maternal‐embryo gas and nutrient exchange begins.^[^ ^17^ ^]^ Thus, elevated glucose levels during early embryogenesis are unlikely to cause elevated ROS at the same time as the key events of organ formation that are disrupted by hyperglycemia.

***Solution***: Clinicians planning using animal models to investigate the causes of birth defects should consult developmental biologists to identify the best strategy for determining the molecular origins of these defects.

A poor understanding of human development also devalues some epidemiological studies. In general, epidemiological studies of birth defect risk factors include exposures during any stage of pregnancy. However, as discussed above, developing embryos are only likely to be vulnerable to environmental teratogens in the first trimester. Once the critical developmental period is past, exposure to a teratogen will have no effect. Thus, studies which include exposures at any time during pregnancy may dilute any signal, reducing the statistical power of the study. Ideally, further restriction of the exposure window for each particular type of birth defect would maximize the chances of detecting a teratogenic effect, although this may be technically challenging.

***Solution***: Prior to designing epidemiological studies, epidemiologists should consult developmental biologists to identify the critical period during pregnancy when the embryo is likely to be most vulnerable to exposure to an environmental teratogen.

## WHAT THE EPIDEMIOLOGIST WISHES THE DEVELOPMENTAL BIOLOGIST KNEW

Epidemiological research is conducted in real‐life situations, not under controlled conditions. Therefore, study design is complicated, and is critical to the success of the study. There many different ways of performing epidemiological studies, and each has distinct advantages and disadvantages. For example, the best quality data comes from studies where the data is collected prospectively, specifically for that study. Here, participants are recruited into the study, and thus data can be collected in a uniform and standardized way. However, this is costly and studies may take years. It also requires participants time and engagement over a long period of time, and thus runs the risk of individuals dropping out of the study. Retrospective data collection is easier and quicker, and can take advantage of existing data sources such as electronic health care records, employment records, and social care data; as well as participant surveys and interviews. However, some of this latter data can be subject to recall bias. This is because the key events of embryogenesis occur in the first 8 weeks of pregnancy. ∼ 50% of pregnancies are unintended,^[^ ^9^ ^]^ and in many cases these events have occurred before the mother is aware she is pregnant. However, diagnosis of a birth defect does not usually occur until prenatal scans at 20 weeks or perhaps not even until birth. This means that an accurate determination of maternal physiology or exposures to potential teratogens several months before may be difficult.

***Solution***: Clinicians or developmental biologists should not attempt an epidemiological study without an expert collaborator. To maximize the chances of generating reliable and useful results, they need to be very carefully designed to fit the research question under investigation. This requires taking into account a wide variety of factors, including whether a cohort or case‐control study would be more appropriate, data source quality, minimizing selection and information bias, and choosing an appropriate statistical analysis.

## CONCLUSION

Exposure to teratogens conditions in utero remains an important cause of human birth defects globally. Understanding how these environmental factors cause birth defects is important. This is because there is great potential to significantly reduce the prevalence of birth defects globally, through changes to influence public health policy, as demonstrated by the remarkable success of folic acid supplementation in reducing NTDs. To do this more efficiently, I believe that closer communication between clinician, epidemiologists and developmental biologists is now required to efficiently harness the advances in molecular and “big data” techniques to increase our understanding of the environmental causes of birth defects, and potentially reduce their global burden.

## CONFLICT OF INTEREST

No conflicts of interest, financial or otherwise, are declared by the author.
